# Caffeinated Drinks and Physical Performance in Sport: A Systematic Review

**DOI:** 10.3390/nu13092944

**Published:** 2021-08-25

**Authors:** Sergio L. Jiménez, Javier Díaz-Lara, Helios Pareja-Galeano, Juan Del Coso

**Affiliations:** 1 Centre for Sport Studies, Universidad Rey Juan Carlos, Fuenlabrada, 28943 Madrid, Spain; 2 Department of Nutrition, Food Science and Physiology, Faculty of Pharmacy and Nutrition, Universidad de Navarra, 31009 Pamplona, Spain; fjdiazl@unav.es; 3 Department of Physical Education, Sport and Human Movement, Autonomous University of Madrid, 28049 Madrid, Spain; helios.pareja@uam.es

**Keywords:** caffeine, exercise performance, elite athlete, side effect, adenosine

## Abstract

Caffeine (1,3,7-trimethylxanthine) is one of the most common substances used by athletes to enhance their performance during competition. Evidence suggests that the performance-enhancing properties of caffeine can be obtained by employing several forms of administration, namely, capsules/tablets, caffeinated drinks (energy drinks and sports drinks), beverages (coffee), and chewing gum. However, caffeinated drinks have become the main form of caffeine administration in sport due to the wide presence of these products in the market. The objective of this systematic review is to evaluate the different effects of caffeinated drinks on physical performance in various sports categories such as endurance, power-based sports, team sports, and skill-based sports. A systematic review of published studies was performed on scientific databases for studies published from 2000 to 2020. All studies included had blinded and cross-over experimental designs, in which the ingestion of a caffeinated drink was compared to a placebo/control trial. The total number of studies included in this review was 37. The analysis of the included studies revealed that both sports drinks with caffeine and energy drinks were effective in increasing several aspects of sports performance when the amount of drink provides at least 3 mg of caffeine per kg of body mass. Due to their composition, caffeinated sports drinks seem to be more beneficial to consume during long-duration exercise, when the drinks are used for both rehydration and caffeine supplementation. Energy drinks may be more appropriate for providing caffeine before exercise. Lastly, the magnitude of the ergogenic benefits obtained with caffeinated drinks seems similar in women and men athletes. Overall, the current systematic review provides evidence of the efficacy of caffeinated drinks as a valid form for caffeine supplementation in sport.

## 1. Introduction

Caffeine (1,3,7-trimethylxanthine) is one of the most common substances used by elite athletes before and during competition [1,2], likely due to its well-supported ergogenic properties in a multitude of exercise scenarios [3]. Since the removal of caffeine from the list of banned substances by the World Anti-doping Agency in 2004, athletes can use caffeine foods and caffeine-containing dietary supplements in any quantity and form without the burden of being sanctioned due to contravening any anti-doping rule. Additionally, caffeine is a socially accepted drug, and its performance-enhancing properties have been recently endorsed by international sports organizations such as the International Olympic Committee [4] and the Australian Institute of Sport [5]. All these factors have increased this stimulant’s presence in the market in the form of dietary supplements for sportspeople and the interest of exercise practitioners. Current recommendations to use caffeine in sport suggest the convenience of consuming moderate doses of caffeine (3–6 mg·kg^−1^) because this dosage is enough to produce benefits to physical performance [6,7] with a low prevalence and magnitude of side effects [8]. On the other hand, the use of high doses of caffeine may be toxic and produce several acute health problems such as cardiovascular events [9].

In sports science research, the traditional form of oral caffeine administration has been through capsules/tablets, although the ergogenic effect of caffeine has also been studied using coffee [10], energy drinks [11], caffeinated sports drinks [12], or chewing gum [13]. The use of caffeinated gum increases the rate of absorption of caffeine [14], but all forms of oral administration of caffeine produce peak caffeine concentration within 0.5 to 2 h after ingestion [15]. Hence, for most sport and exercise situations, the selection of caffeine supplementation forms responds to the athlete’s preference, readiness to obtain the product in the market, and the taste of the caffeine-containing product rather than physiological reasons. In the sport setting, the usage of caffeine-containing drinks may offer some practical benefits over capsules/tablets, namely, quickness of taking a moderate dose of caffeine before exercise and the easier accessibility of caffeinated drinks due to the wide commercial presence of energy drinks in the market. In addition, there are potent advertising campaigns by energy drink manufacturers that are directly aimed at increasing the usage of energy drinks in athletes, which increases the tendency of athletes to use caffeinated drinks as a form of caffeine supplementation. In fact, caffeinated drinks have become one of the most common ways to consume caffeine among young [16] and elite athletes [17]. Energy drinks are beverages that contain carbohydrates, taurine, and group B vitamins, in addition to other less common ingredients such as electrolytes and herbal extracts [18]. However, the ingredient of primary interest is caffeine because, in addition to its ergogenic benefits, it may have the potential to increase mental focus and activeness and it affects the perceptions of energy [19,20]. Due to the moderate concentration of carbohydrates and the lack of electrolytes, energy drinks are habitually consumed before exercise, with the main purpose of providing caffeine at a concentration of ~32 mg·100 mL^−1^ for most drinks. Although less frequent in the market, there are also sports drinks with caffeine with potential ergogenic benefits [21]. In this case, these drinks are designed to provide an amount of caffeine during exercise and to combine caffeine supplementation and rehydration in the same product. They contain an appropriate concentration of carbohydrates to facilitate gastric emptying and the most common electrolytes lost by sweating [22]. To date, there have been reviews focused on the effect of caffeinated drinks on several aspects of physical fitness [23,24] or performance in team sports [11]. However, there is no manuscript addressing the performance-enhancing effect of caffeinated drinks in sport, depending on the characteristics of the discipline. Therefore, this systematic review aims to evaluate the different effects of caffeinated drinks on physical performance in various sports categories, such as endurance, power-based sports, team sports, and skill-based sports.

## 2. Materials and Methods

### 2.1. Search Strategy and Selection of Studies

The Preferred Reporting Items for Systematic Reviews and Meta-Analyses (PRISMA) guideline was followed to conduct and identify the studies to be included as part of the current systematic review [25]. The databases used for the search were PubMed, Web of Science, Medline Complete, and Embase. The keywords used were (“energy drinks” [mesh] OR “energy drinks” OR “energy drink” OR beverages [mesh] OR beverages OR beverage) AND (caffeine [mesh] OR caffeine OR “1,3,7 trimethylxanthine” OR “1,3,7-trimethylxanthine”) AND (“athletic performance” [mesh] OR “athletic performance” OR “athletic performances” OR “sport performance” OR “sports performances” OR sports [mesh] OR team sports OR sport OR athletic OR athletics OR exercise [mesh] OR exercises OR “physical exercise” OR physical exercises OR “isometric exercises” OR “isometric exercise” OR “aerobic exercises” OR “aerobic exercise” OR “plyometric exercise” OR “plyometric training” OR “circuit-based exercise” OR “resistance training” OR “resistance exercise” OR “resistance exercises” OR “endurance training” OR “endurance exercise” OR “endurance exercises” OR running OR walking OR sprint OR sprints OR “repeated sprints” OR “jump training” OR “jump exercise” OR “jump exercises” OR “strength training” OR “strength exercise” OR “strength exercises” OR “physical conditioning” OR “concurrent training”). All original full-text articles in English or translated to English, published from January 2000 to August 2020, were considered for the review. In order to increase the quality of the current review, we only examined peer-reviewed literature and excluded unpublished results in the form of master’s theses, doctoral dissertations, and conference abstracts. 

All of the studies found in the mentioned databases were assessed for duplicates. All duplicates were removed. A two-stage screening process was applied for the selection of the articles. In the first step, all the titles and abstracts were evaluated following the inclusion and exclusion eligibility criteria to identify relevant research for the current review. In the second step, full-text articles were reviewed to determine if they met the optimal inclusion criteria and had significance according to the objective of our current research. The selection of the studies was made following PICOS model eligibility criteria, which categorizes P as population, I as the intervention, C as comparators, and O as the outcomes as well as the study design [26]. For all search articles, the following inclusion criteria were applied to select studies: (a) participants for the included studies must be both males and females within 12 to 60 years of age; (b) all participants must present a healthy status, with no history of cardiovascular disease or serious musculoskeletal conditions; (c) all participants must be categorized as athletes; (d) the intervention of the studies must contain either a chronic and/or acute intake of a caffeine-containing drink (energy drinks or caffeinated sports drinks); (e) the experimental design should be cross-over, and the trials must be blinded and randomized; (f) the ingestion of the caffeine-containing drink must be compared to an identical experimental situation without caffeine; (g) the experiment must contain information regarding the administered caffeinated drink (relative dose of caffeine per kg of body mass, absolute dose of caffeine, or caffeine concentration in the drink); (h) the studies must combine the ingestion of a caffeine-containing drink, with a testing protocol that assesses the physical performance associated to a sport discipline. Even though there is not a specific criterion for the type of physical performance under evaluation, studies that fall under the following sports categories were included in the current review as long as they assessed a parameter of the athlete’s performance: endurance, power, mixed-metabolism team-sports, mixed-metabolism individual sports, and skill-based sports. Additionally, investigations performed with samples from multiple sports were also included in the pool of investigations included in the current analysis. All the study designs must fall under primary studies, including randomized controlled trials, cross-over, cohort, case-control, case reports, and case series. 

Studies were excluded under the following criteria: (a) those studies with a secondary design such as meta-analysis, systematic reviews, and narrative reviews; (b) animal studies; (c) studies conducted on injured or ill participants; (d) studies in which the study sample was not composed of athletes; (e) studies in which the caffeinated drink was administered with other supplements; (f) studies with no placebo/control condition; (g) articles with no full-text available; (h) studies that used magnitude-based inferences (MBI) to infer an ergogenic effect of caffeinated drinks, as this type of statistical approach may increase the likelihood of Type 1 and Type 2 errors [27]. Opinion pieces, commentaries, and editorials were not considered for inclusion. 

**Figure 1:**
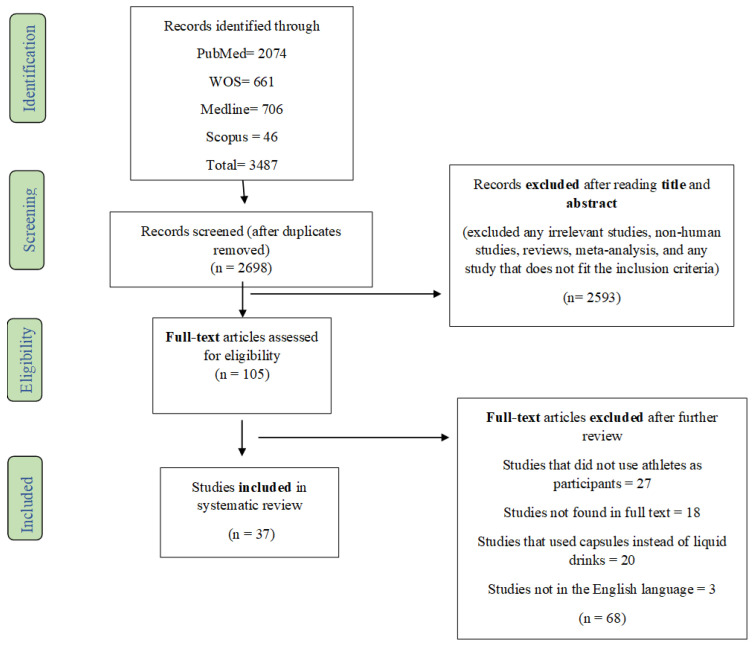
Flow diagram of literature search according to the PRISMA statement.

A total of 3487 studies were initially obtained through the different databases. From this total, 789 were identified as duplicates and removed. Of that number, 2593 studies were excluded after the first screening of title and abstract as it was determined that they did not meet eligibility criteria standards. Afterward, 105 studies were evaluated by full-text reading to certify that they meet all eligibility inclusion and exclusion criteria. From the 105 studies, an additional 68 studies were removed due to the following reasons: 27 studies used physically active individuals as participants; 18 studies were not available in full text; 20 studies used capsules/tablets as a form of caffeine administration instead of caffeinated drinks; 3 studies were not in English or were not translated to English. Finally, the total number of studies included in the current systematic review was 37 (Figure 1). 

### 2.2. Data Extraction

From the selected studies, specific pieces of information were extracted, such as author’s name, year of publication, participants’ characteristics (number of participants, age, gender, study design, sports discipline, supplementation protocol (the type of caffeinated drink, dose of caffeine, and timing of administration), exercise performance testing, and main outcome result observed in the studies.

### 2.3. Quality Assessment and Risk of Bias

The level of quality for each of the selected studies was assessed following the Cochrane Collaboration Guidelines [28]. The Cochrane Risk of Bias tool for randomized clinical studies was used. This tool is focused on seven domains to assess the studies’ bias: sequence generation and allocation concealment (selection bias), blinding of participants and personnel (performance bias), blinding of outcome assessment (detection bias), incomplete outcome data (attrition bias), selective reporting (reporting bias), and other sources of bias (other bias). The risk of bias of each study was categorized as low, high, or unclear for each of the seven domains. 

## 3. Results

### 3.1. Quality Assessment and Risk of Bias

**Figure 2:**
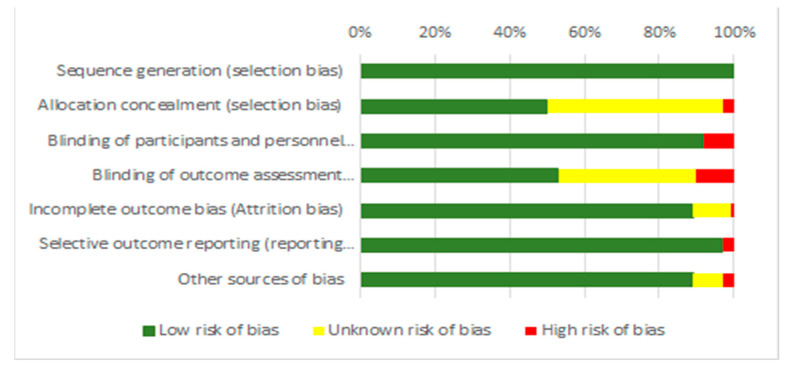
Risk of bias summary.

According to the quality and risk of bias assessment (Table 1 and Figure 2), the following information was obtained: all 37 studies included in the systematic review were categorized as low risk of bias in the “random sequence generation” category. In the allocation concealment category, 1 study was considered high risk, 18 studies were an unclear risk, and 18 different studies were considered low risk. Regarding the blinding of participants and personnel (performance bias), 3 studies (single-blind) were considered high risk, and 34 studies (double-blind) were defined as low risk. Additionally, 4 studies were considered at high risk for the blindness of outcome assessment (detection of bias), 14 studies as unclear risk, and 19 were noted as low risk. In regard to the category of “incomplete outcome data”, only 1 study was considered high risk, 4 studies were considered unclear risks, and 32 studies fell under a low risk of bias. Moreover, 1 study was considered high risk in terms of selective outcome reporting, and the remaining 36 studies were considered low risk. Lastly, a single study was assigned to have an overall high risk of bias, 4 studies were considered to have unclear risk overall, and 32 studies were determined to have a low risk of bias. This information is detailed in Table 1 and Figure 2.

### 3.2. Description of Included Studies

From the final number of studies included in the current systematic review, 34 studies used double-blind experimental designs while the remaining 3 used single-blind designs. Taking all the studies together as a whole, the total sample consisted of 692 participants (416 males, 208 females, and 68 undetermined), with an age range from 12 to 45 years old. All studies used healthy, active athletes from different sports disciplines (6 studies on cycling, 4 on soccer, 4 on distance running, 3 on volleyball, 2 on swimming, 2 on rugby-sevens, 2 on badminton, 2 on tennis, 2 on golf, 2 on football, 1 on fencing, 1 on rugby, 1 on field hockey, 1 on basketball, and 2 on multiple sports). Regarding habituation to caffeine, the samples used diverse methods to categorize daily caffeine intake. From the final number of studies included in the current systematic review, 10 used participants with moderate habituation to caffeine, with different levels of daily caffeine intake; 10 used participants categorized as light caffeine users; 2 used participants with low daily caffeine intake; and 1 study used a sample with several categories of daily caffeine intake (namely, non-caffeine users vs. low-to-moderate users vs. high-caffeine users). The remaining 14 studies did not provide information to categorize the participants’ habituation to caffeine. 

In 16 of the studies, the caffeine content in the energy drink was administered as an absolute dose, ranging from 75 to 215 mg, while the remaining 21 studies used a relative dose of caffeine relativized by participant’s body mass, ranging from 1.3 to 7.5 mg·kg^−1^. The caffeinated drinks were administered 10 to 15 min before exercise in 2 studies, 30 to 40 min before exercise in 5 studies, 50 to 60 min before exercise in 34 studies and 90 min before exercise in 1 study. The remaining five studies administered the caffeinated drinks at the beginning of the trial as well as during the trial. From the total, 30 studies used taste-matched placebo drinks/beverages, 5 studies used non-caffeinated beverages with a different taste to the caffeinated trial drinks, and, in 2 investigations, the information on drinks’ taste was not reported. Only 16 studies used a post-trial questionnaire to obtain information to ascertain if participants correctly guessed in which trial they had received the caffeinated drink. In all investigations, *p*-values were used to determine the existence of an ergogenic effect with the administration of the caffeinated drink, but 12 studies also calculated any form of effect size to gauge the magnitude of the ergogenic effect. In 5 studies, the time between experimental trials was between 5 and 6 days; in 29 studies, it was set to one week between trials, and, in 3 studies, the time between trials was one week or more. The information on the participants, study design, sport, supplementation protocol and caffeine dose, and main performance outcome has been detailed in Appendix A. 

## 4. Discussion

In the following section, we present a discussion of the main findings of the current systematic review by using the physiological characteristics of the sport to categorize each investigation: endurance-based sports, power-based sports, individual and team sports with mixed aerobic–anaerobic metabolic contributions, skill-based team sports, and investigations with a sample of athletes of multiple sports. 

### 4.1. Endurance-Based Sports

In the current systematic review, 11 studies were characterized as endurance-based sports (Appendix A). Out of the six studies that examined cycling, five reported a significant improvement in the tests performed with the ingestion of caffeinated drinks over the placebo trial [12,21,35,47,57]. In contrast, one study did not report any performance benefit with the ingestion of an energy drink [54]. Hulston and Jeukendrup [35] reported a reduction in the time to complete a ~45-min cycling time-trial using a caffeinated glucose drink consumed before and during an exercise bout that preceded the time-trial when testing 10 male cyclists. Talanian and Spriet [21] also reported a cycling performance benefit when using two caffeinated sports drinks over placebo using a similar protocol that included rehydration with the different drinks during steady-state exercise followed by a ~30-min time trial. Cureton et al. [12] also reported that adding caffeine to a sports drink increased performance during a ~15-min cycling time trial after exercising at moderate intensity. The benefit obtained in these cycling trials was similar to data reported in studies by Ivy et al. [47] and Quinlivan et al. [57]. In these latter studies, the authors used an energy drink to provide caffeine before exercise, and there was no steady-state exercise performed before the cycling time trial. Reported outcomes suggest that a caffeinated drink performance benefit may be obtained with both sports drinks and energy drinks. However, it seems that caffeinated sports drinks are more applicable to exercise scenarios with longer duration, where the drinks are used for both rehydration and caffeine supplementation during a sector with lower intensity to obtain benefits in the latter phases of exercise (namely, a cycling stage). 

On the other hand, energy drinks may be more appropriate to provide caffeine before exercise in shorter events in which it is crucial to exert maximal intensity from the beginning of exercise (namely, a cycling time trial). Of note, it is essential to note that the addition of a small amount of caffeine to a sports drink may limit the benefits of the caffeinated sports drink on cycling performance [21], especially if the dose ingested with the drink is under 3 mg·kg^−1^. Four studies used distance runners as their participants, but only Prins et al. [56] found a significant improvement in the 5-km treadmill running time with the ingestion of an energy drink. The remaining three studies by Schubert et al. [51], Rica et al. [59], and Sheehan and Hartzler [60] did not report any significant performance benefit with respect to a placebo trial. Again, dosage may be behind the differences between investigations as, in the study by Prins et al. [56], the dose of caffeine was double that of the other studies (160 vs. 80 mg). The remaining study in this category was conducted by Arazi et al. [31], who reported a significant time decrease in the 100-m crawl velocity test using an energy drink in a group of young female swimmers. Overall, the data of these investigations indicate that usage of caffeinated drinks is a beneficial strategy to enhance endurance performance if the amount of drink is enough to provide a dose close to, or above, 3 mg of caffeine per kg of body mass.

### 4.2. Power-Based Sports

Only two of the studies included in the current review were conducted in power-based sports. Hoffman et al. [46] tested 12 male strength and power athletes provided with an energy drink. These authors reported a significant increase in reaction performance following consumption of the energy drink, although the caffeinated beverage did not produce any change over three repetitions of the 20-s Wingate anaerobic performance test. In the study reported by Lara et al. [49], the authors documented a significant improvement in countermovement jump height, maximal force during the handgrip test, and reduced time during a 50-m simulated swimming competition with the ingestion of an energy drink. The lack of further studies performed in strength/power athletes precludes us from obtaining solid evidence about the ergogenicity of caffeinated drinks on this type of sport. However, the well-supported ergogenic role of caffeine in strength- and power-based exercise [63,64,65,66] suggests that caffeinated drinks may be a useful supplementation protocol to increase muscle performance in short-time and high-intensity exercise if they are ingested before exercise and provide a minimum ergogenic dose of caffeine.

### 4.3. Team Sports

Fourteen studies were included in the category of team sports, all of them using energy drinks as the source of caffeine supplementation. In four studies, soccer players were used as the study sample. Del Coso et al. [37] reported that the pre-exercise ingestion of a caffeinated energy drink increased jump height, ability to perform repeated sprints, and total and high-intensity running distance during a simulated game in a group of male soccer players. Using a similar protocol in terms of supplementation and testing, Lara et al. [48] reported a significant increase in jump height, average peak running velocity during a sprint test, and total and high-intensity running distance during a simulated game in a group of female soccer players. The two other studies that examined soccer players were conducted by Astorino et al. [32] and Carvajal and Moncada [33]. Both reported no significant differences in any soccer-specific tests performed. Again, as it happened in endurance-based exercise, the dose of caffeine provided with the drinks may be responsible for the differences between investigations, as clear performance benefits on soccer performance were reported when the administered caffeine dose equaled 3 mg·kg^−1^ [37,48] while a lack of effect was documented when the dose equaled ≤2 mg·kg^−1^ of caffeine [32,33]. Interestingly, the effect of pre-exercise caffeine energy drink intake, documented in soccer players, was similar to the ones reported in male rugby players [38], female rugby-sevens players [39,55], and field hockey players [41]. In contrast, Woolf et al. [62] did not report any ergogenic benefit of energy drink intake in a sample of football players. Gwacham and Wagner [45] indicated that in their study sample, the athletes who did not regularly consume much caffeine (i.e., low caffeine consumers) were more likely to benefit from an energy drink than those who were regular caffeine users, as they documented that habituation to caffeine was a confounding factor in the ergogenic benefit obtained after acute caffeine intake. This is supported in the literature as a progressive tolerance to the ergogenic benefits of caffeine has been documented with chronic caffeine intake [67,68]. Collectively, these outcomes suggest that energy drinks may be a performance-enhancing supplementation strategy for players with low daily caffeine intake, while their effectiveness may be reduced in players with habituation to caffeine. In this regard, and from a practical perspective, it may be recommended to reduce the daily consumption of caffeine in players habituated to this stimulant when planning to use acute caffeine intake as an ergogenic aid. 

Three of the studies included in this category were performed on volleyball players. Del Coso et al. [40] reported that in 15 male volleyball players, there was a significant increase in ball velocity in a spike test and in jumps during different jump tests when the participants were supplemented with an energy drink before exercise. Additionally, the energy drink also increased the number of successfully performed volleyball actions over the placebo trial, as rated by a volleyball coach blinded to the treatments. Similar results were documented by Pérez-López et al. [50], who experimented with similar protocols in a sample of female volleyball players. The third study on volleyball players was conducted by Fernández-Campos et al. [43] and reported no significant differences between the energy drink and the placebo drink, likely because the dose provided in this investigation was ~2 mg·kg^−1^. Regarding the study on basketball players [29], it seems that caffeinated drinks may also enhance single and repeated jumping performance, with potential further benefits for basketball players if the dose of caffeine administered is at least 3 mg·kg^−1^ [69]. Overall, the intake of an energy drink to provide 3 mg·kg^−1^ of caffeine may be helpful to enhance high-intensity and power-based actions (namely, sprints, jumps, high-intensity running) in team sports. This may be crucial for team sports performance as these actions, and the capacity to repeat them after short recovery periods, are connected with the most decisive situations in team sports [70,71]. Lastly, the use of similar time-motion analysis techniques in ecological environments (namely, real and simulated competitions) employed in several investigations in this section indicates that caffeinated energy drinks increased team sport-specific performance to a similar extent in both men and women athletes.

### 4.4. Individual Sports with Mixed Aerobic–Anaerobic Metabolism

In the current systematic review, four studies were categorized as individual sports with mixed aerobic–anaerobic metabolism. Two studies were conducted on badminton players; Abian et al. [30] investigated the effect of a caffeinated energy drink on 16 male badminton players and reported a significant increase in power and height achieved during sport-specific jumps, in addition to a higher number of body impacts during a simulated match. Similarly, Clarke and Duncan [36] reported a significant improvement in serve accuracy and sprinting action and a reduction in anticipation time when using a caffeinated sports drink ingested before and during a fatiguing exercise protocol. The other two studies used tennis players as their study sample. Gallo-Salazar et al. [44], in young male and female tennis players, reported a significant increase in the running pace at high-intensity and in the number of sprints during a simulated match with the pre-exercise ingestion of an energy drink. Although it did not attain statistical significance, energy drink usage tended to increase the overall number of points won over the placebo, especially when the player was at serve. Peltier et al. [53] reported that male tennis players had a significantly higher stroke frequency during match play, in addition to decreased ratings of perceived exertion, when caffeinated sports drinks were consumed before and during a simulated tournament that included three matches on two consecutive days. Collectively, the support conclusions provided in this section suggest that the ingestion of caffeinated drinks may have benefits in some sports-specific skills in racket sports for both high-intensity running and sport-specific gestures such as serving and strokes. 

### 4.5. Skill-Based Sports

Three studies were determined to fit this category, as these disciplines’ primary performance outcomes are associated with accuracy. Two of these studies used golf players, and one used fencing athletes as their study sample. Mumford et al. [52] reported a significant improvement in total score, greens in regulation, and drive distance with the ingestion of an energy drink before and after 9 holes during each 18-hole round. Stevenson et al. [61] reported improved golf performance during putting and increased feelings of alertness in golfers when they had ingested a caffeinated sports drink before and during simulated competition. In fencers, during a simulated practice, Doyle et al. [42] reported significant increases in response time, accuracy, and arousal when an energy drink was consumed before exercise. Caffeinated energy drinks can be ergogenic in sports with a high level of technical demands, where concentration and focusing are crucial. Although the number of studies included in this section is low, the performance improvements reported in these studies may be of interest to athletes in skill-based sports. It is likely that the effects reported in this type of sport are mainly related to the caffeine-induced changes to the central nervous system through the inhibition of the adenosine-fatiguing effect [72], leading to increased excitability of neuronal tissues, level of arousal, and cognition [73].

### 4.6. Multiple Sports

Three studies included in this systematic review used athletes from several different sports disciplines. Chen et al. [34] examined the effect of an energy drink on a sample of multi-sport athletes and reported a significant increase in isometric muscle strength during a maximal voluntary contraction. As in other investigations referred to above, the ergogenic effect of the caffeinated drink on muscle power and muscle endurance did not show a gender bias because both male and female athletes obtained benefits of similar magnitude [34]. Rahnama et al. [58] observed greater VO_2max_ data and times to exhaustion during running on a treadmill when the participants ingested two types of energy drinks, at least when compared to a placebo. Lastly, Salinero et al. [8] documented that athletes of different sports reported an increased self-perceived muscle power during exercise as well as a higher prevalence of side effects after exercise, as well as insomnia, nervousness, and activeness when they had ingested a caffeinated energy drink to increase performance. Overall, the studies reviewed indicate that the ergogenic benefits of caffeine are obtained even when using heterogeneous samples of athletes, reinforcing the wide capacity of caffeine to enhance exercise performance. 

## 5. Conclusions

The current systematic review, which included 37 studies examining the effect of caffeinated drinks on physical performance in different sports disciplines, suggests that both sports drinks with caffeine and energy drinks effectively increase several aspects of sports performance. Evidence supports the ergogenic role of caffeinated drinks on endurance-based sports, power-based sports, individual and team sports, and skill-based sports. However, their efficacy is linked to the need to ingest an amount of drink that provides at least 3 mg of caffeine per kg of body mass. Due to their composition, caffeinated sports drinks are useful for consumption during long-duration exercise, where the drinks are used for both rehydration and caffeine supplementation to increase performance in the latter phases of the exercise. However, energy drinks may be more appropriate to provide caffeine before exercise. Lastly, the magnitude of the ergogenic benefits obtained with caffeinated drinks seems similar in women and male athletes. Although it has not been the focus of the current review, it is essential to note that caffeinated drinks may produce several side effects, which should be evaluated when planning to use this type of drink to increase sports performance.

## Figures and Tables

**Table 1:** Quality assessment and risk of bias.

Study	Random Sequences Generation (Selection Bias)	Allocation Concealment (Selection Bias)	Blinding of Participants/Researchers (Performance Bias)	Blindness of Outcome Assessment (Detection Bias)	Incomplete Outcome Data (Attrition Bias)	Selective Outcome Reporting (Reporting Bias)	Other Sources of Bias
Abian et al., 2014 [29]	**+**	**?**	**+**	**+**	**+**	**+**	**+**
Abian et al., 2015 [30]	**+**	**?**	**+**	**+**	**+**	**+**	**+**
Arazi et al., 2016 [31]	**+**	**+**	**+**	**+**	**+**	**+**	**+**
Astorino et al., 2012 [32]	**+**	**+**	**-**	**-**	**+**	**-**	**+**
Carvajal and Moncada, 2005 [33]	**+**	**+**	**+**	**+**	**+**	**+**	**+**
Chen et al., 2015 [34]	**+**	**?**	**+**	**+**	**+**	**+**	**+**
Hulston and Jeukendrup, 2008 [35]	**+**	**?**	**+**	**+**	**+**	**+**	**+**
Clarke and Duncan, 2016 [36]	**+**	**?**	**+**	**+**	**+**	**+**	**+**
Cureton et al., 2007 [12]	**+**	**?**	**+**	**+**	**-**	**+**	**+**
Del Coso et al., 2012 [37]	**+**	**+**	**+**	**+**	**+**	**+**	**+**
Del Coso et al., 2013 [38]	**+**	**+**	**+**	**+**	**+**	**+**	**+**
Del Coso et al., 2013 [39]	**+**	**+**	**+**	**+**	**?**	**+**	**+**
Del Coso et al., 2014 [40]	**+**	**+**	**+**	**+**	**+**	**+**	**+**
Del Coso et al., 2016 [41]	**+**	**+**	**+**	**+**	**+**	**+**	**+**
Doyle et al., 2016 [42]	**+**	**?**	**+**	**?**	**+**	**+**	**?**
Fernández-Campos et al., 2015 [43]	**+**	**?**	**+**	**?**	**?**	**+**	**+**
Gallo-Salazar et al., 2015 [44]	**+**	**+**	**+**	**?**	**+**	**+**	**+**
Gwacham and Wagner, 2012 [45]	**+**	**?**	**+**	**+**	**+**	**+**	**+**
Hoffman et al., 2009 [46]	**+**	**?**	**+**	**?**	**+**	**+**	**+**
Ivy et al., 2009 [47]	**+**	**?**	**+**	**?**	**+**	**+**	**+**
Lara et al., 2014 [48]	**+**	**+**	**+**	**+**	**?**	**+**	**+**
Lara et al., 2015 [49]	**+**	**+**	**+**	**+**	**+**	**+**	**+**
Perez-Lopez et al., 2015 [50]	**+**	**+**	**+**	**+**	**+**	**+**	**?**
Schubert et al., 2013 [51]	**+**	**+**	**-**	**?**	**+**	**+**	**+**
Mumford et al., 2016 [52]	**+**	**+**	**+**	**+**	**+**	**+**	**?**
Peltier et al., 2013 [53]	**+**	**?**	**+**	**-**	**+**	**+**	**?**
Phillips et al., 2014 [54]	**+**	**+**	**-**	**?**	**+**	**+**	**+**
Portillo et al., 2017 [55]	**+**	**+**	**+**	**+**	**+**	**+**	**+**
Prins et al., 2016 [56]	**+**	**?**	**+**	**?**	**+**	**+**	**+**
Quinlivan et al., 2015 [57]	**+**	**-**	**+**	**?**	**+**	**+**	**+**
Rahnama et al., 2010 [58]	**+**	**?**	**+**	**?**	**+**	**+**	**+**
Rica et al., 2019 [59]	**+**	**?**	**+**	**?**	**?**	**+**	**-**
Salinero et al., 2014 [8]	**+**	**+**	**+**	**?**	**+**	**+**	**+**
Sheehan and Hartzler, 2011 [60]	**+**	**+**	**+**	**?**	**+**	**+**	**+**
Stevenson et al., 2009 [61]	**+**	**?**	**+**	**?**	**+**	**+**	**+**
Talanian and Spriet, 2016 [21]	**+**	**?**	**+**	**?**	**+**	**+**	**+**
Woolf et al., 2009 [62]	**+**	**?**	**+**	**?**	**+**	**+**	**+**

**+** Low risk of bias. **?** Unclear risk of bias. **-** High risk of bias.

## Data Availability

No new data were created or analyzed in this study. Data sharing is not applicable to this article.

## Appendix A

**Table A1:** Investigations in endurance-based sports disciplines.

Study	Participants	Habituation to Caffeine	Study Design	Sport	Supplementation	Dose	Main Outcome
Arazi et al. (2016) [31]	36 females 14 ± 1 years	Not reported	DB, R, Cx	Swimming	Big Bear energy drink or placebo, consumed 15 min before exercise	78 mg	Time decrease in a 100 m crawl velocity test
Hulston and Jeukendrup (2008) [35]	10 males 27 ± 7 years	All subjects were identified as caffeine users to varying extent (186 ± 101 mg·day^−1^, range = 70–400 mg·d^−1^)	DB, R, Cx	Cycling	Glucose drink with or without caffeine or placebo, consumed at the start and during exercise	5.3 mg·kg^−1^	Performance improvement in a time trial
Cureton et al. (2007) [12]	16 males 28 ± 7 years	Habitually caffeine users (150 ± 113 mg·day^−1^, range = 9–482 mg·day^−1^)	DB, Cx	Cycling	Sports drink with caffeine (C + C) and without caffeine (CHO) or placebo, consumed before exercise	195 mg·L^−1^	Greater mean total work performed during a 15 min ride for the C + C trial
Ivy et al. (2009) [47]	6 males and 6 females 27 ± 2 years	Not reported	DB, R, Cx	Cycling	Red Bull energy drink or placebo consumed 40 min before exercise	160 mg	Performance improvement in a time trial
Schubert et al. (2013) [51]	6 males 23 ± 2 years	Mean daily caffeine intake of 80 mg·day ^−1^	SB, R, Cx	Running	Yerba MatéOrganic Energy Shot (YM), Red Bull Energy Shot (RB), or a placebo, consumed 1 h before exercise	YM = 140 mg, RB = 80 mg	No significant difference in time-trial performance
Phillips et al. (2014) [54]	11 males 33 ± 9 years	Between 80 and 350 mg·day^−1^	SB, R, Cx	Cycling	Red Bull energy drink (RB), Cola drink (CD), or placebo, consumed 50 min before exercise	RB = 160 mg, CD = 160 mg	No significant difference in a simulated road race
Prins et al. (2016) [56]	13 males and 5 females 20 ± 3 years	Noncaffeine users (*n* = 1), low-to-moderate–caffeine users (50–200 mg·day^−1^; *n* = 13) and high-caffeine users (>200 mg·day^−1^; *n* = 4)	DB, R, Cx	Running	Red Bull energy drink or placebo, consumed 1 h before the trial	160 mg	Improvement in a 5-km treadmill running time
Quinlivan et al. (2015) [57]	11 males 32 ± 6 years	Regular caffeine consumers (mean caffeine consumption 295 mg·day^−1^)	DB, R, Cx	Cycling	Red Bull energy drink (RB), anhydrous caffeine (AC), or placebo, consumed 90 min before the trial	RB = 3 mg·kg^−1^,AC = 3 mg·kg^−1^	Significant improvement in time to complete a cycling trial
Rica et al. (2019) [59]	55 males and females 33 ± 6 years	Not reported	DB, R, Cx	Running	Energy drink or a placebo, consumed 1 h before exercise	80 mg	No significant improvement in the Cooper test
Sheehan and Hartzler (2011) [60]	9 males and 3 females 21 ± 2 years	Not reported	DB, R, Cx	Running	Cherry Blast XS^®^ energy drink or a placebo, consumed 30 min before the trial	80 mg	No significant increase in VO_2max_
Talanian and Spriet (2016) [21]	11 males and 4 females 23 ± 1 years	Only one subject was a habitual caffeine user (>50 mg·day^−1^)	DB, R, Cx	Cycling	Two caffeinated carbohydrate-electrolyte drinks (CE1 and CE2) or a placebo, consumed during exercise	CE1 = 100 mg, CE2 = 200 mg	Performance improvement in a cycling time trial

DB = double-blinded, SB = single-blinded, R = randomized, Cx = crossover.

**Table A2:** Investigations in power-based sports disciplines.

Study	Participants	Habituation to Caffeine	Study Design	Sport	Supplementation	Dose	Main Outcome
Hoffman et al. (2009) [46]	12 males 21 ± 1 years	Not reported	DB, R, Cx	Strength/power athletes	Redline Extreme energy drink or placebo, consumed 10 min before exercise	125 mg	Increase in reaction performance
Lara et al. (2015) [49]	14 males 20 ± 3 years	Light caffeine consumers (less than one can of soda or energy drink per day)	DB, R, Cx	Sprint swimming	Fure, ProEnergetics energy drink, or placebo, consumed 1 h before exercise	3 mg·kg^−1^	Increased performance in a 50-m simulated swimming competition

DB = double-blinded, R = randomized, Cx = crossover, VO_2max_ = maximum oxygen uptake.

**Table A3:** Investigations in mixed-metabolism team sports.

Study	Participants	Habituation to Caffeine	Study Design	Sport	Supplementation	Dose	Main Outcome
Abian et al. (2014) [29]	16 males 15 ± 1 years	Light caffeine consumers (<60 mg·day^−1^)	DB, R, Cx	Basketball	Fure, ProEnergetics energy drink, or placebo, consumed 1 h before exercise	3 mg·kg^−1^	Increase in repeated jump performance
Astorino et al. (2012) [32]	15 females 20 ± 1 years	Twelve of 15 women were current caffeine consumers, although intake ranged from 1 to 7 days/week	SB, R, Cx	Soccer	Red Bull energy drink or placebo, consumed 1 h before the trial	1.3 mg·kg^−1^	No significant increase in repeated sprint performance
Carvajal and Moncada (2005) [33]	20 males 20 ± 2 years	All participants were moderate coffee consumers (2–4 cups per day)	DB, Cx	Soccer	Energy drink, no drink, or placebo, consumed 30 min before exercise	132 mg	No significant differences in strength and velocity
Del Coso et al. (2012) [37]	19 males 21 ± 2 years	Not reported	DB, R, Cx	Soccer	Sugar-free Red Bull energy drink or placebo, consumed 1 h before the trial	3 mg·kg^−1^	Higher high-intensity running during a simulated game
Del Coso et al. (2014) [40]	15 males 22 ± 7 years	Light caffeine consumers (<30 mg·day^−1^)	DB, R, Cx	Volleyball	Fure, ProEnergetics energy drink, or placebo, consumed 1 h before exercise	3 mg·kg^−1^	Improved performance in several jump tests
Del Coso et al. (2013) [39]	16 females 23 ± 2 years	Light caffeine consumers (<60 mg·day^−1^)	DB, R, Cx	Rugby-sevens	Fure, ProEnergetics energy drink, or placebo, consumed 1 h before exercise	3 mg·kg^−1^	Higher high-intensity running during a simulated game
Del Coso et al. (2016) [41]	13 males 23 ± 4 years	Not reported	DB, R, Cx	Field hockey	Fure, ProEnergetics energy drink, or placebo, consumed 1 h before exercise	3 mg·kg^−1^	Higher high-intensity running during a simulated game
Del Coso et al. (2013) [38]	26 males 25 ± 2 years	Light caffeine consumers (<60 mg·day^−1^)	DB, R, Cx	Rugby	Fure, ProEnergetics energy drink, or placebo, consumed 1 h before exercise	3 mg·kg^−1^	Higher high-intensity running during a simulated game
Fernández-Campos et al. (2015) [43]	19 females 22 ± 5 years	Not reported	DB, R, Cx	Volleyball	Energy drink, no drink, or placebo, consumed 30 min before exercise	110 mg	No significant difference in any performance variable
Gwacham and Wagner (2012) [45]	20 males 20 ± 2 years	Not reported	DB, R, Cx	Football	AdvoCare Spark energy drink or placebo, consumed 1 h before trial	120 mg	No significant improvement in sprint performance
Lara et al. (2014) [48]	18 females 21 ± 2 years	Light caffeine consumers (not more than one coffee or one serving of energy drink per day)	DB, R, Cx	Soccer	Fure, ProEnergetics energy drink, or placebo, consumed 1 h before exercise	3 mg·kg^−1^	Higher high-intensity running during a simulated game
Pérez-López et al. (2015) [50]	13 females25 ± 5 years	Not reported	DB, R, Cx	Volleyball	Fure, ProEnergetics energy drink, or placebo, consumed 1 h before exercise	3 mg·kg^−1^	Higher number of body impacts during a simulated game
Portillo et al. (2017) [55]	13 females 23 ± 2 years	The daily consumption of caffeine was low (60 mg·day^−1^)	DB, R, Cx	Rugby-sevens	Fure, ProEnergetics energy drink, or placebo, consumed 1 h before exercise	3 mg·kg^−1^	Increase in the number of body impacts during real competition
Woolf et al. (2009) [62]	17 males 20 ± 2 years	Not reported	DB, R, Cx	Football	Energy drink or placebo, consumed 1 h before the trial	5 mg·kg^−1^	No significant difference in any performance variable

DB = double-blinded, SB = single-blinded, R = randomized, Cx = crossover.

**Table A4:** Investigations in mixed-metabolism individual sports.

Study	Participants	Habituation to Caffeine	Study Design	Sport	Supplementation	Dose	Main Outcome
Abian et al. (2015) [30]	16 males 25 ± 7 years	Light caffeine consumers (<60 mg·day^−1^)	DB, R, Cx	Badminton	Fure, ProEnergetics energy drink, or placebo, consumed 1 h before exercise	3 mg·kg^−1^	Higher performance in badminton-specific jumps
Clarke and Duncan (2016) [36]	12 males 28 ± 9 years	Not reported	DB, R, Cx	Badminton	Carbohydrate drink (CHO), caffeinated drink (ED), a drink containing carbohydrate and caffeine (C + C), or placebo, consumed before and during exercise	ED = 4 mg·kg^−1^, C + C = 4 mg·kg^−1^	Significant improvement in serve accuracy and sprinting actions
Gallo-Salazar et al. (2015) [44]	10 males and 4 females 16 ± 1 years	Light caffeine consumers (less than 1 can of soda or energy drink per day)	DB, R, Cx	Tennis	Fure, ProEnergetics energy drink, or placebo, consumed 1 h before exercise	3 mg·kg^−1^	Higher number of sprints during a simulated match
Peltier et al. (2013) [53]	8 males 26 ± 6 years	Moderate caffeine consumers (1–2 cups of coffee or equivalent per day)	DB, R, Cx	Tennis	Caffeinated sports drinks or placebo, consumed before and during a simulated tournament	215 mg	Higher stroke frequency during play

DB = double-blinded, R = randomized, Cx = crossover.

**Table A5:** Investigations in skill-based sports.

Study	Participants	Habituation to Caffeine	Study Design	Sport	Supplementation	Dose	Main Outcome
Doyle et al. (2016) [42]	13 males and females 18–23 years	Not reported	DB, R, Cx	Fencing	Energy drink or placebo consumed 1 h before exercise	1.5–7.5 mg·kg^−1^	Increases in response time and accuracy during a fencing-specific test
Mumford et al. (2016) [52]	12 males 35 ± 14 years	Not reported	DB, R, Cx	Golf	Energy drink or placebo, consumed before and after 9 holes during each 18-hole round	155 mg	Improvements in total score, greens in regulation, and drive distance
Stevenson et al. (2009) [61]	20 males 23 ± 4 years	Habitual caffeine users, consuming 157 ± 47 mg·day^–1^	DB, R, Cx	Golf	Lucozade Caffeine Boost energy drink or placebo, consumed right before the trial and at holes 6 and 12	1.6 mg·kg^−1^	Improved putting performance

DB = double-blinded, R = randomized, Cx = crossover.

**Table A6:** Investigations in multiple sports.

Study	Participants	Habituation to Caffeine	Study Design	Sport	Supplementation	Dose	Main Outcome
Chen et al. (2015) [34]	10 males/19 females 20 ± 2 years	All participants were not regular caffeine consumers (<200 mg·week^−1^)	DB, R, Cx	Tennis, basketball, and soccer	Energy drink or placebo, consumed 1 h before the exercise	6 mg·kg^−1^	Significant increase in isometric muscle strength
Rahnama et al. (2010) [58]	10 males 22 ± 2 years	All participants were moderate caffeine users	DB, R, Cx	Not specified	Red Bull energy drink (RB), Hype energy drink (HE), or placebo, consumed 40 min before exercise	RB = 85 mg, HE = 75 mg	Greater VO_2max_ and time to exhaustion
Salinero et al. (2014) [8]	53 males/37 females 24 ± 6 years	All participants were light caffeine consumers (<60 mg·day^−1^)	DB, R, Cx	Rugby, volleyball, tennis, badminton, swimming, soccer, and hockey	Fure, ProEnergetics energy drink, or placebo, consumed 1 h before exercise	3 mg·kg^−1^	Increase in self-perceived muscle power during exercise

DB = double-blinded, R = randomized, Cx = crossover, VO_2max_ = maximum oxygen uptake.
